# Measures of recovery used in clinical settings among people with opioid use disorder: a rapid review of the literature

**DOI:** 10.1186/s13722-026-00700-3

**Published:** 2026-07-28

**Authors:** Kelly W. Gagnon, Margaret Baldwin, Yahya Alnashri, Jessica Jaiswal, Karen Cropsey, Ellen Eaton

**Affiliations:** 1 Center for Community Health and Intervention Research, Department of Psychiatry and Behavioral Medicine, Medical College of Wisconsin, Milwaukee, WI USA; 2 Heersink School of Medicine, University of Alabama at Birmingham, Birmingham, AL USA; 3 Department of Family & Community Medicine, College of Medicine, Prince Sattam Bin Abdulaziz University, Al-Kharj, Saudi Arabia; 4 Department of Health Policy & Organization, School of Public Health, The University of Alabama at Birmingham, Birmingham, AL USA; 5 Department of Family and Community Medicine, University of Alabama at Birmingham, Birmingham, AL USA; 6 Center for Interdisciplinary Research on AIDS, Yale University School of Public Health, New Haven, CT USA; 7 Department of Psychiatry and Behavioral Neurobiology, Heersink School of Medicine, University of Alabama at Birmingham, Birmingham, AL USA; 8 Division of Infectious Diseases, Department of Medicine, Heersink School of Medicine, University of Alabama at Birmingham, Birmingham, AL USA

**Keywords:** Opioid use disorder, Recovery measures, Substance use treatment, Abstinence, Recovery capital, Self-efficacy

## Abstract

**Background:**

The United States (US) has observed increasing rates of opioid use and overdose deaths since the 1990s. For patients receiving medications for opioid use disorder (OUD), clinical visits are a critical touchpoint to evaluate progress towards recovery.

**Objective:**

This rapid review aims to identify psychometrically validated measures of recovery used in clinical settings in the US for individuals with OUD.

**Methods:**

A systematic search of Embase and Health and Psychosocial Instruments databases was conducted. Inclusion criteria: peer-reviewed studies in English from 2000 to 2023 that applied recovery measures within U.S. clinical treatment settings for individuals with OUD. References were screening and data extracted using Rayyan. Identified measures were mapped to patient-centered treatment goals.

**Results:**

19 studies met the inclusion criteria, including 37 validated measures of recovery. Measures were predominantly designed to be self-administrated. Measures fell under three domains: exclusively measuring drug use, measuring drug use and other factors, and exclusively measuring factors other than drug use. The most used measure was the Addiction Severity Index, used in 10 studies. After mapping to established patient-centered treatment goals, avoid illicit drug use (*n* = 14, 38%) was the construct identified most frequently across included measures.

**Conclusion:**

This rapid review of OUD recovery measures found a wide range of measures used over the study period. The most used measures focused on frequency and severity of drug use. Patient-centered treatment goals were largely covered by identified measures. Self-administer design of most measures presents opportunity to leverage patient-reported outcomes in clinical settings to evaluate recovery and inform treatment plans. Future research should explore how customizable PROMs and PREMs for patients in treatment for OUD can support goal tracking, improve patient-clinician decision-making, and help patients with recovery.

**Clinical trial number:**

Not applicable.

## Introduction

Since the late 1990s, the United States (US) has seen a continual increase in the prevalence of opioid use and related deaths. In 2019, an estimated 7 million individuals had an opioid use disorder (OUD) in the US [1]. In 2023, over 81,000 deaths occurred due to overdose involving opioids, which constitutes more than 75% of all drug overdose deaths that year and a ten-fold increase in opioid-related overdose deaths since 1999 [2, 3]. In the same year, 100,000 emergency department visits for nonfatal opioid overdose were reported by the Centers for Disease Control and Prevention [4, 5].

Fortunately, OUD is one of the few substance use disorders (SUD) with evidence-based pharmacotherapy options. Medications for opioid use disorder (MOUD), such as the opioid agonists buprenorphine and methadone and the antagonist naltrexone, are effective in treating opioid use disorder and reducing harms across multiple health outcomes [6–8]. As part of medication management, patients on MOUD attend regular medical appointments to assess maintenance and receive/refill medications [9–11]. At these appointments, patients discuss their progress and may receive complementary services, like counseling [9–11]. These visits are critical check points not only for medication management, but also for tracking progress towards treatment goals [9–11].

The general goal of treatment is to assist patients in achieving recovery. The Substance Abuse and Mental Health Services Administration defines recovery as “a process of change through which individuals improve their health and wellness, live a self-directed life, and strive to reach their full potential” [12]. In alignment with this definition, the American Society of Addiction Medicine recommends approaching addiction treatment using a patient-centered chronic care model [13]. This guidance includes establishing treatment goals based on current needs, designing individualized treatment timelines, and aiming for progression towards less intensive services [13].

A recent systematic review of patient-reported treatment goals found 12 main treatment domains, including treatment retention, avoiding illicit drugs, reducing criminal activity, achieving stability and normalcy, gaining education and employment, and improving familial and social relationships [14]. Given the holistic scope of treatment and recovery goals from the perspective of people with OUD, it is important for clinical providers to account for these goals as part of individualized treatment plans.

An important component of medical visits for MOUD management is the assessment of treatment effectiveness and progression towards established goals [15, 16]. Validated measures are psychometrically evaluated tools to assess latent factors that align with treatment goals [17]. Validated measures are useful to clinicians and patients who want to document progress quantitatively, complementary to biological measures, as well as qualitatively via visit notes [18, 19]. These measures may also support clinicians and patients utilizing a patient-centered chronic care model by providing outcomes data to inform timelines and intensity of services [20, 21].

While validated measures related to patient-reported treatment goals exist, it is not yet known which measures have been implemented or utilized in clinical settings [22]. This rapid review aimed to address this dearth of research by examining published, peer-reviewed studies that utilized one or more validated measure of recovery. The goal of this work was to summarize published measures that have been utilized in clinical settings in the US among people with OUD and the alignment of these measures with patient-centered treatment goals. The results of this review will demonstrate gaps in recovery measurement and potential avenues for improving treatment for OUD via consideration other measures related to patient-centered treatment goals.

## Review question

- What psychometrically evaluated measures of recovery from opioid use disorder are being used in studies occurring in clinical settings in the United States and US territories?

## Methods

As there is not yet a consensus on specific methods for the rapid review process, we utilized a common approach for our review as found in the existing literature [23]. The PRISMA-COSMIN for Outcome Measurement Instrument was used as guidance for writing this review [24].

### Review criteria and scope

This rapid review aimed to identify measures of recovery from opioid use disorder that have been used in clinical settings in the US. In alignment with this aim, the criteria for inclusion are listed in Table 1.

**Table 1:** Inclusion criteria

Category	Criteria
Population	Persons with opioid use disorder
Context	Clinical substance use treatment settings in the United States and US territories
Key Variables	Measure of recovery from opioid use disorder
Sources	Empirical peer-reviewed journal articles, published in English between 2000–2023

### Population and context

This review focused on persons with opioid use disorder who were engaged with a substance use treatment program. This includes office-based addiction treatment clinics, opioid treatment programs, other outpatient SUD clinics, and detoxification or rehabilitation facilities. Participants did not need to receive medications for opioid use disorder to meet the inclusion criteria for this review. There were no sociodemographic exclusion criteria; participants could be of any age, gender, race, socio-economic status, etc. All studies had to occur in clinical settings in the US, as policies and treatment vary country to country.

### Outcomes

The outcome of interest for this review was measures of recovery for opioid use disorder. As the definition of recovery is relative and iterative, we included any measure utilized by authors to represent recovery. Thus, measures could range from topics such as abstinence and opioid cravings to self-efficacy and quality of life. In anticipation of some or most studies not providing a definition of recovery, we included any and all measures the authors’ chose to assess recovery, treatment progress, and/or treatment effectiveness. To assist with determining which measures authors considered relevant and related to recovery, we included measures that were selected as primary or secondary outcome variables or key independent variables (i.e., not covariates). Measures had to be psychometrically evaluated to be included, whether they were instruments or single item measures. Given some recovery constructs of interest are latent in nature, we focused on measures that have demonstrated validity and reliability in assessing constructs of interest.

### Sources

This rapid review focused on empirical, full text articles in peer-reviewed journals. Literature reviews, case studies, commentaries, conference proceedings, and other publications, including grey literature, were excluded. Additionally, articles had to be in English and published between 2000 and 2023.

#### Search strategy

Embase (embase.com) and Health and Psychosocial Instruments (EBSCOhost) electronic databases were searched May 19, 2023, from inception of each database via a librarian-assisted database search. We used keywords related to opioid disorder treatment instruments. The reproducible searches for both databases are available in Appendix I. A total of 165 citations were retrieved. Duplicates were removed by the librarian manually and using EndNote’s duplicate identification feature. Since reference extraction occurred from Embase and the Health and Psychosocial Instruments databases, many references were extracted with the measure name with multiple measures (and references) per study. Thus, additional duplicates were identified in abstract and full text screening so that articles were only included once.

### Selection process

**Fig. 1:**
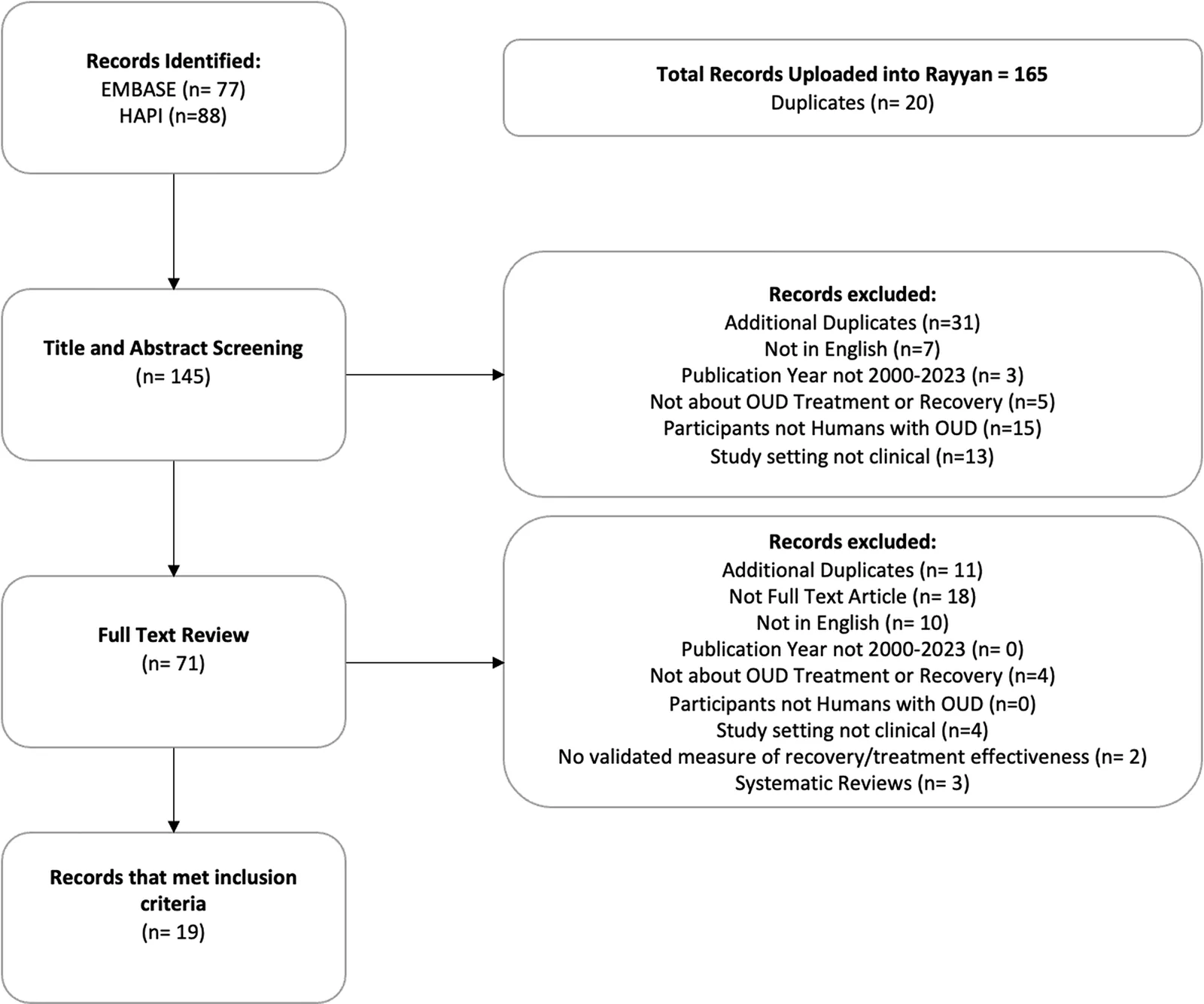
PRISMA diagram of inclusion and exclusion of records

Records extracted from search sources were collated and uploaded into Rayyan (Rayyan Systems, Inc., Cambridge, MA) [25]. The “Detect Duplicates” function was used in Rayyan to review and eliminate duplicate records. As stated above, our selection process was chosen from a summary of most common practices for rapid reviews. Specifically, titles and abstracts were screened for inclusion by one reviewer. These inclusion decisions were then checked by a second reviewer, including records marked undetermined by the first reviewer. Full text articles were then aggregated and uploaded into Rayyan to complete full text review. The same review process was used for full text articles to finalize inclusion. At both stages, any records that were marked undetermined by both reviewers were discussed during research team meetings, where principal investigators and study coordinators were present, for resolution. This process resulted in six articles that met the inclusion criteria for this rapid review. A PRISMA diagram outlining the review process, including inclusion and exclusion, can be found in Fig. 1 [26].

### Data collection process

The data extraction tool within Rayyan was used to collect data. Reviewer one extracted relevant data points and reviewer two compared the included full text articles to the preliminary data extraction chart to identify any missing or incorrect elements. Any necessary additions and corrections were made before concluding the data collection process. Any discrepancies or uncertainties were discussed at research team meetings.

### Data items

In alignment with common rapid review practices, data items extracted during the data collection process were limited to those that directly addressed the questions of the review. We focused on collecting key characteristics of each study and measure of recovery. A full summary of data items can be found in Table 2.

**Table 2:** Summary of data items extracted

Category	Data Item
Study Characteristic	Study Title
	Year Published
	Study Location
	Study Population
Recovery Measure Characteristic	Measure Name
	Number of Items
	Response Structure
	Collection Modalities

### Data analysis

To determine the extent to which measures being used in clinical settings align with patient-centered treatment goals, we mapped selected measures to the list of 12 patient-centered treatment goals from Sanger et al.’s recent systematic review (2022) [14]. These included: treatment retention, avoid illicit drugs, avoid withdrawal, reduce criminal activity, mental health improvement, stability and normalcy, taper off MOUD, improve housing, education and employment, and improved familial/ social relationships. To map measures to these goals, we looked at the constructs of focus for each measure and measure items to identify alignment between measures and patient-centered treatment goals. Given the length and scope of some measures, measures could be mapped to multiple goals. Microsoft Excel (Microsoft Corporation, Redmond, Washington) was used to calculate the sum and proportion of each measure by patient-centered treatment goal and year.

## Results

### Study characteristics

19 studies met the inclusion criteria for this rapid review (Table 3) [27–45]. While studies were identified over the course of the inclusion period, more studies were published in the latter half of the timeframe, with three and five published in 2021 and 2022, respectively. Studies were slightly geographically diverse with most occurring on the east coast and in the northeastern region of the US. In alignment with the inclusion criteria, all studies took place in clinical substance use treatment settings, including outpatient opioid treatment programs, methadone clinics, and rehabilitation units. Study populations ranged in size from 13 to 543.

**Table 3:** Study characteristics

Study Title	Year	Study Location and Sample Size	Measures Used
Efficacy of Contingency Management and Significant Other Involvement	2002	127 patients recently discharged from detoxification in New Haven, CT	Addiction Severity Index, Substance Abuse Calendar, Urine Drug Screening
A Pilot Double Blind Placebo Controlled Trial of Sertraline with Naltrexone in the Treatment of Opiate Dependence	2002	13 patients with OUD on naltrexone treatment in New Haven, CT	Obsessive-compulsive Opiate-craving Scale, Beck Depression Inventory, Urine and Blood Screening
Recovery from opioid addiction in DATOS	2003	432 patients from 96 drug treatment programs in 11 cities in the US	Recovery Perceptions Scale, Addiction Severity Index, Urine and Hair Analysis
Millon Clinical Multiaxial Inventory–III Subtypes of Opioid	2004	125 patients with OUT in outpatient detoxification in New Haven, CT	Substance Use Calendar, Addiction Severity Index, Beck Depression Inventory, Urine Drug Testing
Predictors of Outcome in LAAM, Buprenorphine, and Methadone Treatment for Opioid Dependence	2005	165 patients with OUD in Maryland	Addiction Severity Index, Beck Depression Inventory, Urine Drug Testing
Methadone maintenance improves cognitive performance after two months of treatment	2006	17 patients with OUD at a methadone maintenance program in Boston, MA	Addiction Severity Index, Urine Drug Testing
The relationship of social support to treatment entry and engagement: The Community Assessment Inventory	2010	196 patients with OUD in Baltimore, Maryland	Community Assessment Inventory, Addiction Severity Index
Impact of depression on abstinence self-efficacy and substance use outcomes among emerging adults in residential treatment	2012	302 patients in addiction treatment programs in Minnesota	Inventory of Drug Use Consequences, Form 90D, Alcohol and Drug Use Self-Efficacy
Buprenorphine treatment for probationers and parolees	2015	64 probationers and parolees in outpatient treatment programs in Baltimore	Addiction Severity Index, Urine Drug Testing
The effect of bundling medication-assisted treatment for opioid addiction with mHealth: Study protocol for a randomized clinical trial	2016	440 patients on medication assisted treatment for OUD from 3 addiction treatment centers in Wisconsin	Timeline Follow back, Brief Addiction Monitor, Satisfaction with Life Scale, Morisky Medication Adherence Scale, Treatment Self-Regulation Questionnaire, Drug-taking Confidence Questionnaire, Self-stigma in Substance Abuse Scale, Urine Drug Screen
Engagement in online pain self-management improves pain in adults on medication-assisted behavioral treatment for opioid use disorders	2018	60 patients with chronic pain with OUD in medications for addiction treatment in Washington, US	Current Opioid Misuse Measure, PHQ-9, GAD-7
Envisioning a future: Values clarification in early recovery from opioid use disorder	2021	93 in residential treatment for OUD in the Northeastern US	Addiction Severity Index, Valuing Questionnaire, Commitment to Sobriety Scale, Delay Discounting Task
The Genomics of Opioid Addiction Longitudinal Study (GOALS): study design for a prospective evaluation of genetic and non-genetic factors for development of and recovery from opioid use disorder	2021	400 patients receiving medications for OUD in New Jersey	PHQ-9, GAD-7, Addiction Severity Index
Three-month outcomes from a patient-centered program to treat opioid use disorder in Iowa, USA	2021	40 patients in OUD treatment program in Iowa	Brief Addiction Monitor, Assessment of Recovery Capital
Six-month outcomes of the HOPE smartphone application designed to support treatment with medications for opioid use disorder and piloted during an early statewide COVID-19 lockdown	2022	25 patients in office-based opioid treatment program in Virginia	Brief Self-control Scale, Drug Abstinence Self-Efficacy Scale, Mental Health Inventory, Perceived Stress Scale, Medical Outcomes and Social Support Survey Instrument, Perceived Stigma of Substance Abuse Scale
Buprenorphine exposure levels to optimize treatment outcomes in opioid use disorder	2022	543 patients with moderate to severe OUD	Timeline Follow back, Opioid Craving VAS, Urine and Blood Testing
Rationale, design, and methodology of a randomized pilot trial of an integrated intervention combining computerized behavioral therapy and recovery coaching for people with opioid use disorder: The OVERCOME study	2022	Study protocol, not yet enrolled. Participants will be patients receiving buprenorphine in South Carolina	Timeline Follow back, Opioid Craving Scale, Addiction Severity Index, Risk Behavior Assessment, PHQ-9, GAD-7, Delay Discounting Task, Recovery Capital Matrix, Health-related Quality of Life, Saliva testing
Improvements in recovery capital are associated with decreased alcohol use in a primary opioid use disorder treatment-seeking cohort	2022	133 medications for addiction treatment clinic patients in Iowa City, Iowa	Assessment of Recovery Capital
Long-term recovery from opioid use disorder: recovery subgroups, transition states and their association with substance use, treatment and quality of life	2022	216 patients with OUD in Virginia	Beck Depression Inventory, Short form Health Survey, WHO Quality of Life Scale- Brief

### Measures of recovery

The 19 included studies in this review comprised 37 distinct validated instruments to measure recovery, as all studies utilized more than one measure (Table 4). All measures were tools that had been psychometrically evaluated and validated. Nearly all measures were designed to be self-administered by the participant. A few studies used additional indicators of recovery in combination with a validated instrument. While these measures were not the primary focus of this review, we extracted them as a secondary datapoint since they were related to our outcome of interest.

**Table 4:** Measure characteristics

	Measure Name	Number of Items	Response Structure	Collection Modalities
1	Addiction Severity Index	161	Various	Interviewer
2	Assessment of Recovery Capital	50	Agree/disagree	Self
3	Brief Addiction Monitor	17	Likert	Self or Interviewer
4	Delay Discounting Task	6	7 delays	Self
5	Substance Use Calendar	7	Calendar	Self
6	Timeline Followback	Varies	Calendar	Self or Interviewer
7	Risk Behavior Assessment	Varies	Various	Interviewer
8	Commitment to Sobriety Scale	5	Likert	Self
9	Current Opioid Misuse Measure	17	Likert	Self
10	Drug-taking Self-Confidence Questionnaire	50	Likert	Self
11	Form 90D	39	Various	Interviewer
12	Inventory of Drug Use Consequences	50	Likert	Self
13	Obsessive-compulsive Opiate Craving	12	Likert	Self
14	Opioid Craving Scale	3	Visual Analog Scale	Self
15	Self-sufficiency Matrix	19	Likert	Interviewer
16	Beck Depression Inventory	21	Likert	Self
17	General Anxiety Disorder-7	7	Likert	Self
18	Patient Health Questionnaire-9	9	Likert	Self
19	Alcohol and Drug Use Self-Efficacy	20	Likert	Self
20	Brief Self-Control Scale	13	Likert	Self
21	Brief Symptom Inventory	53	Likert	Self or Interviewer
22	Community Assessment Inventory	37	Likert	Self
23	Drug Abstinence Self-Efficacy Scale	20	Likert	Self
24	Health Related Quality of Life (EQ-5D-3 L)	5	Likert	Self
25	Medical Outcomes and Social Support Survey	19	Likert	Self
26	Mental Health Inventory	38 or 5	Likert	Self
27	Morisky Medication Adherence Scale	8 or 4	Yes/No and Likert	Self
28	Opioid Craving Visual Analog Scale	1	Visual Analog Scale	Self
29	Perceived Stigma from Substance Abuse Scale	8	Likert	Self
30	Perceived Stress Scale	10	Likert	Self
31	Recovery Perceptions Scale	9	Likert	Self
32	Satisfaction with Life Scale	5	Likert	Self
33	Self-stigma in Substance Abuse Scale	40	Likert	Self
34	Short Form Health Survey	36	Likert	Self
35	Treatment Self-regulation Questionnaire	19	Likert	Self
36	Valuing Questionnaire	10	Likert	Self
37	WHO quality of life scale- Brief	26	Likert	Self

### Summary of measures by overarching domain

After reviewing the 37 measures extracted for this review, three domains were evident: (1) measures that exclusively assess drug use, (2) measures that assess drug use, treatment adherence, and other factors, and (3) measures that exclusively assess other factors.

### Measures exclusively assessing drug use

9 measures exclusively evaluated frequency and/or severity of drug use. The most commonly used measures were the Timeline Followback (*n* = 3) and the Substance Use Calendar (*n* = 2). All other measures that only assessed drug use were used in only one study. Although not the primary aim of this study, it was noted that studies sometimes utilized secondary, biological, measures to assess drug use and treatment adherence. These included urine (*n* = 9), salvia (*n* = 1), blood (*n* = 1), and hair toxicology (*n* = 1). A summary of measures under this domain are as follows:

The Substance Use Calendar, Timeline Followback, and Risk Behavior Assessment were measures used to evaluate patients’ use of opioids and other substances and ascertain important outcomes like recency of use, extent of use, and amount used. The Substance Use Calendar is a self-report instrument inquiring about drug use on a daily basis and allows for the continuous evaluation of substance use [46]. The Timeline Followback is a calendar that assesses daily drug use and can be self-administered, administered on the computer, or by a clinician [47]. Lastly, the Risk Behavior Assessment is a structured interview tool to measure HIV risk, drug use, and sexual activity over the past 30 days [48].

Under the domain of drug use, studies also utilized measures specific to craving as this symptom is among the criteria for OUD [49]. Specifically, Three scales were identified that were specific to craving: (1) Obsessive-Compulsive Opiate Craving Scale [50], (2) Opioid Craving Scale [51, 52], and (3) Opioid Craving Visual Analog Scale [53]. These measures evaluate thoughts about opiates and interference, desire and control, resistance to thoughts and intentions, and extent to which they feel cravings for opioids.

### Measures of drug use, treatment adherence, and other factors

8 measures included constructs and items assessing drug use, treatment, adherence, and other factors. Other factors included social support, income, housing, employment, mental health, physical health, etc. The most commonly used measure under this domain, and across the review, was the Addiction Severity Index (*n* = 10). Form-90D and the Brief Addiction Monitor were used in two studies, with all other measures being found in only one study. A summary of measures under this domain are as follows:

Under this domain, three measures included the evaluation of drug use as well as other potential problem areas. These included the Addiction Severity Index, Form 90-D, and the Brief Addiction Monitor. The Addiction Severity Index is a validated tool that assesses seven potential problem areas: medical (9 items), employment/support status (10 items), alcohol/drug (31 items), legal (26 items), family/social (17 items), and psychological (20 items) [54]. This measure also provides interviewer severity and confidence ratings for each section to allow the interviewers to document their perceptions of the patients’ needs and their belief in the accuracy of responses [55]. Form 90-D is a structured assessment tool that measures frequency of drug use over a 90-day period. This measure also inquires about patients’ medication and adherence, medical encounters, education, religious attendance, and work [46, 56]. The Brief Addiction Monitor is a 17-item tool for patients in treatment for substance use disorders. This measure evaluates risk of relapse and worsening severity, protective behaviors, and substance use [57].

Two measures under this domain were similar in their evaluation of drug use and other factors; however, these measures focused on the presence of factors to support recovery. These included the Assessment of Recovery Capital and the Self-sufficiency Matrix. The Assessment of Recovery Capital is a 50-item list of agree/disagree statements pertaining to multi-dimensional elements of recovery, including having a sense of purpose, free of threat or harm, and recent periods of substance intoxication [58]. A brief version of this measure was also utilized in a study included in this review. The Brief Assessment of Recovery Capital is a 10-item, 6-point Likert scale instrument [59].

Two measures were specific to treatment and their self-efficacy and confidence to maintain treatment: (1) Morisky Medication Adherence Scale and (2) Treatment Self-Regulation Questionnaire. The Morisky Medication Adherence Scale is a self-report tool to evaluate patients’ medication adherence, including inquiring about whether patients’ cease medication when they feel better [60]. The Treatment Self-Regulation Questionnaire evaluates why patients’ are or are not engaging in treatment, including autonomous and controlled regulation [61].

Additionally, this review included a study that utilized the Inventory of Drug Use Consequences to assess past and recent consequences from drug use, including negative mental and physical health and damage to social relationships. Lastly, one measure in this domain was specific to opioid misuse and other factors among patients with chronic pain. The Current Opioid Misuse Measure is a 17 item self-assessment measure to monitor chronic pain patients on opioid therapy [62]. This tool can be used to monitor patients on long-term opioid treatment for aberrant medication-related behaviors as well as track outcomes among patients with chronic pain who also have opioid use disorder.

### Measures exclusively assessing factors other than drug use

Studies included measures related to recovery and treatment effectiveness that did not assess drug use. Measures under this domain evaluated key factors related to recovery, including: mental and physical health, self-efficacy and confidence, social support, life satisfaction and quality, and stigma.

Five measures included in this review were specific to mental and physical health: 1) Beck Depression Inventory [63], General Anxiety Disorder-7 [64], Patient Health Questionnaire-9 [65], Mental Health Inventory [66], and Short Form Health Survey [67]. These were utilized to evaluate changes in participants’ mental and physical health during treatment and recovery.

Five measures under this domain evaluate self-efficacy, self-confidence, and self-control: The Alcohol and Drug Use Self-Efficacy Scale, Drug Abstinence Self-Efficacy Scales, Delay Discounting Task, Drug-Taking Confidence Questionnaire, and Brief Self-Control Scale. The Alcohol and Drug Use Self-Efficacy Scale is a 20-item, 4-point Likert scale assessing patients’ confidence to avoid alcohol and drugs in high-risk situations [53]. Similarly, the Drug Abstinence Self-Efficacy Scale is a 20 item, 5-point Likert instrument evaluating patient’s confidence to avoid drugs in high-risk situations [68, 69] This scale consists of four subscales: Negative Affect, Social/Positive, Physical and other Concerns, and Cravings/Urges [68, 69]. The Delay Discounting Task is a psychological tool that measures impulsivity and has been specifically tested among opioid-dependent individuals [70]. This measure and its outcomes have been shown to be associated with patients’ ability to enact self-control and avoid opioid use [71]. The Drug-Taking Confidence Questionnaire is a 50-item self-report to assess patients’ coping self-efficacy with situation specific examples [72]. Lastly, the Brief Self Control scale is a 10-ten item, 5-point Likert scale instrument on which patients’ self-report the extent to which they feel they can do things like resist temptation and refuse things that are bad for them [73].

Studies included in this review also utilized measures of life quality/satisfaction and whether patients’ felt they were living in alignment with their goals. These measures included: Health-related Quality of Life (EQ-5D-3 L) [74], Recovery Perceptions Scale [75, 76], Satisfaction with Life Scale [77], Valuing Questionnaire [78], and Quality of Life Scale- Brief [79].

Under this domain, this review also found measures related to social support (Community Assessment Inventory [80] and Medical Outcomes and Social Support Survey [81]), stigma (Perceived Stigma of Substance Abuse Scale [82] and Self-stigma in Substance Abuse Scale Self-stigma in Substance Abuse Scale), stress (Perceived Stress Scale [83]), and commitment (Commitment to Sobriety Scale [84]).

### Alignment of measures with patient-centered treatment goals

**Fig. 2:**
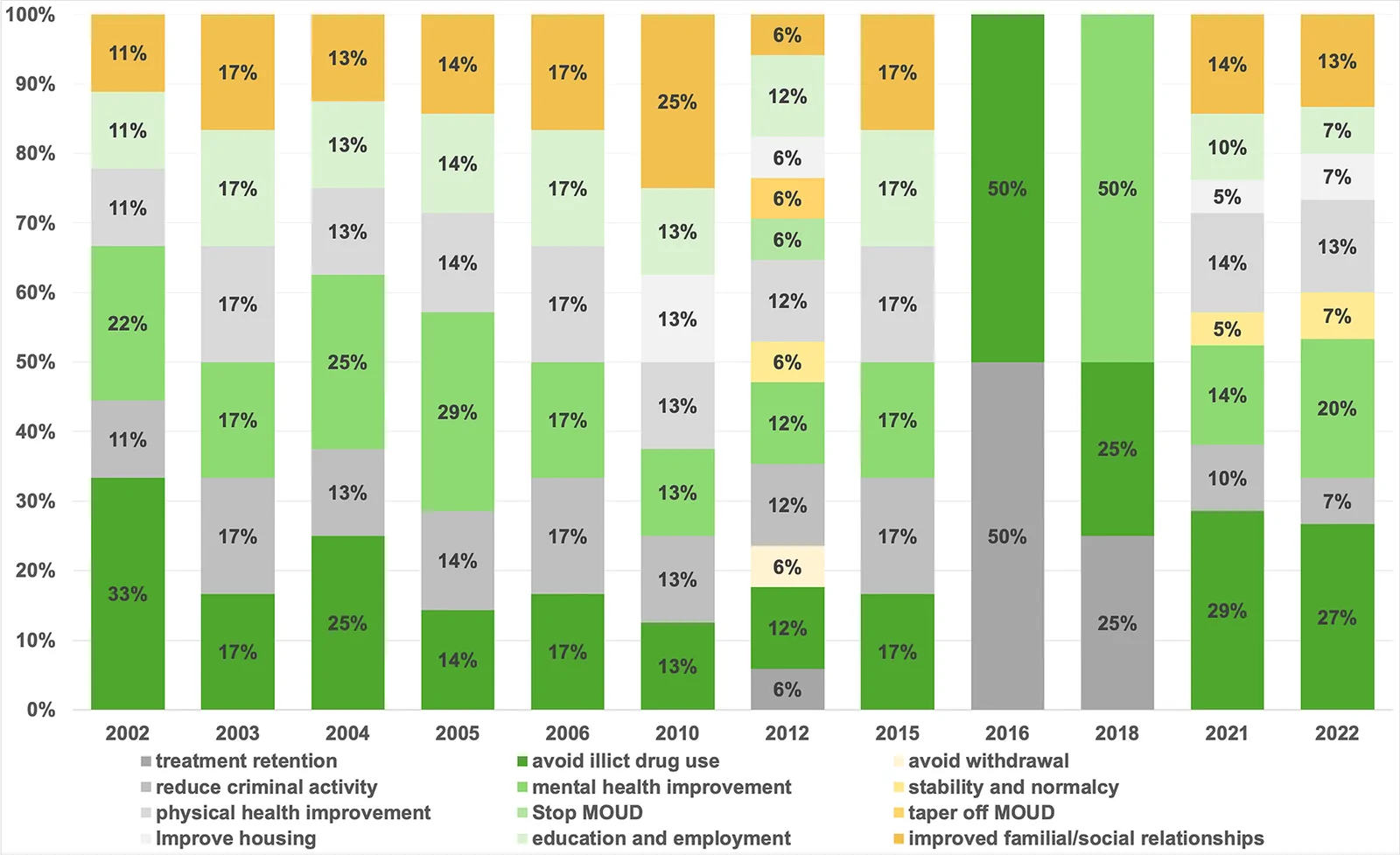
Proportion of patient-centered treatment goals present in measures by year of publication

After mapping measure constructs and items from the final 37 measures to patient-centered treatment goals from Sanger at al. (2022), the most common goal across measures was avoid illicit drug use (*n* = 14, 38%). The second most common goals incorporated into measures were mental health improvement (32%, *n* = 12) and improved familial/social relationships (24%, *n* = 9) Other goals covered in 10–20% of measures, were stability and normalcy (14%, *n* = 5), education and employment (14%, *n* = 5), and Improve housing (11%, *n* = 4). Treatment retention (8%, *n* = 3), avoiding withdrawal (3%, *n* = 1), stop MOUD (3%, *n* = 1), and taper off MOUD (3%, *n* = 1) were goals least present across measures. In an exploration of distribution of patient-centered treatment goal constructs and items by publication year (Fig. 2), an increase in the number of treatment goals covered is observed in the early 2010s and 2020s.

While mapping measures to patient-centered treatment goals, it was observed that some measures included constructs or items that did not map to any of these goals, but to other important factors. These included: self-control and self-efficacy, religious engagement, life satisfaction and quality, stress, stigma, goal achievement and behavioral alignment, and structural factors (safety, mobility, healthcare coverage, etc.).

## Discussion

The purpose of this rapid review was to identify measures of recovery being applied in studies focused on patients with OUD in clinical settings in the US. This review uncovered three domains of measures being used in the literature: (1) measures that exclusively assess drug use, (2) measures that assess drug use, treatment effectiveness, and other factors, and 2) measures that exclusively assess other factors. Generally, identified measures mapped to all patient-centered treatment goals from the literature, with a few exploring additional goals and outcomes (e.g., self-efficacy and quality of life). The most common patient-centered goals present in identified measures were avoiding illicit drug use, improving mental health, and improving physical health. The findings from this study provide insight into the measures most often associated with treatment and recovery from OUD and opportunities for improving assessment.

A primary finding of this review was that the most frequently used measures were focused on assessing frequency and severity of drug use and mental and physical health. The vast majority of identified measures, encompassing all other patient-centered treatment goals, were only used once across included studies. This resulted in minimal coverage of patient-centered treatment goals like reducing criminal activity, stability and normalcy, and improve housing. However, some included measures fell outside of the patient-centered treatment goals from Sanger et al.’s 2002 systematic review [14]. These measures covered constructs such as self-efficacy and confidence, quality and satisfaction with life, goal and value alignment, stress, and stigma. Other investigations into patient-important measures of MOUD treatment success identified similar goals, including being happy, feeling neat and clean, optimism, self-worth, and handling frustrations [85]. This pilesorting study among patients found that not using opioids was ranked with higher importance over decreasing use of opioids [85]. The results of this review demonstrate the acknowledged importance of measuring drug use as a patient-centered treatment goal, whether their goal is to decrease or abstain from use. The breadth and diversity of measures identified in this review also align with the evident complexity of recovery and need for consideration of factors other than drug use and mental and physical health that are important to patients.

A secondary finding of this study is that most identified measures were designed to be self-administered. This presents a promising opportunity for the implementation of patient-reported outcome measures (PROMs) in clinical settings to evaluate treatment effectiveness and recovery and inform shared decision making between clinicians and patients. Previous literature has elucidated distinct types of PROMs, differentiating origin, value, and relevance of PROMs to patients [86]. This literature highlights the importance of utilizing PROMs that were developed in collaboration or with the input of patients and selecting measures that align with their values [86]. Of note, it can be challenging to identify the original study that describes the development of measures, presenting a barrier to the selection of measures that are patient-generated or centered. While ‘patient-irrelevant’ PROMs may be specific to organizational or research priorities, it is critical to acknowledge their lack of alignment with patients and their care [86]. Given the complexity, iterative, and personal nature of recovery, it may be best to select measures for PROMs based on patients’ treatment goals. This approach would facilitate tracking progress towards patients’ goals and could be incorporated into discussions about medication management and treatment planning. However, implementation of PROMs is challenging, with multi-level documented barriers like challenges with technology, competing demands, and limited time [87, 88]. Despite known barriers, overcoming challenges to implementation is likely a worthwhile effort in pursuit of patient-centered care as PROMs have been documented to improve communication, prioritize patient concerns, and amplify their voices in shared decision-making [88]. To support selection of PROMS, clinicians and patients could select from a comprehensive list of patient-reported outcome measures for recovery from opioid use disorder found in a recently published systematic review [22].

An important consideration in pursuit of patient-centered care to support recovery from OUD is the inclusion of patient reported experience measures (PREMs). Unlike PROMs that evaluate patient outcomes, PREMs assess patients’ experience in care and can include measures of relational or functional components of care [89, 90]. These measures can provide critical insight into whether patients feel they are being treated with respect, are experiencing stigma, and the extent to which they feel they are involved in decisions about their care [90]. Another consideration is the need to minimize both patient and clinician burden. This includes thoughtful selection of PROMs and PREM and use of other clinical measures as it aligns with patients’ treatment plans. There are treatment goals that logically are best measured without validated tools. As found in this review, the least common goals detected in measures were treatment retention and stop and taper MOUD. While some studies did track treatment retention, authors did not elect to utilize validated measures, instead using variables such as follow-up attendance. Similarly, the authors likely did not elect to use measures specific to stopping or tapering MOUD, instead using prescription data. In some cases, prescription data were supplemented with patient self-report of medication adherence and urine, blood, hair, or saliva testing. Using multi-modal data points from patients’ health records can reduce documentation burden and allow for the inclusion of PROMs specific to patients’ non-medical goals.

We acknowledge that this review has its limitations. First and foremost, given the scope of the rapid review, we explored which measures are being used in studies of people with OUD in clinical settings. A larger systematic or scoping review of measures being used across and by all substances is warranted. Additionally, we placed a focus on psychometric databases as our source. As such, it is inevitable that other databases contain studies pertaining to recovery measures in clinical substance use treatment settings that would have been applicable to this review. A more appropriate method to identify all psychometric measures being used in these settings would be a scoping or systematic review, rather than the narrow focus provided by our rapid review. Second, our review excluded measures associated with the frequency of opioid use. Finally, as noted in the methods, there is not a single definition of recovery, so our review encompasses proxies for recovery, including treatment effectiveness and progression, that were otherwise not directly entitled “Recovery”.

## Conclusion

Overall, this review identified a wide range of measures of recovery being used among patients with OUD in clinical settings in the US. While the mostly frequently used measure and patient-centered treatment goal of focus pertained to reducing illicit drug use, it is evident from this review that there are many options for measures of recovery from OUD that could be leveraged to support clinical decision making with patients on MOUD. In addition to future studies exploring the extent to which these and other measures were patient-generated or centered, research should explore how customizable PROMs and PREMs for patients in treatment for OUD can support goal tracking, improve patient-clinician decision-making, and help patients with recovery.

## Data Availability

The datasets used and/or analyzed during the current study are available from the corresponding author on reasonable request.

## Appendix I. Search strategy

Search conducted on May 22, 2023.

**Table:**

Database	Search Terms	Items Found
Embase	(measur*:ab, ti OR scale*:ab, ti OR psychometric*:ab, ti) AND recover*:ab, ti AND (((opioid OR opiate) NEAR/2 (addiction* OR disorder)): ti)	77
Health and Psychosocial Instruments (HaPI)	(((opioid OR opiate) N2 (addiction* OR disorder))) AND (treatment* OR therap* OR recover* OR rehab*)	88

## Abbreviations

US: United States
OUD: Opioid Use Disorder
SUD: Substance Use Disorders
MOUD: Medications for Opioid Use Disorder
PROM: Patient Reported Outcome Measure
PREM: Patient Reported Experience Measure
